# On being stuck: the pandemic crisis as affective stasis

**DOI:** 10.1007/s11097-022-09855-1

**Published:** 2022-10-01

**Authors:** Fabian Bernhardt, Jan Slaby

**Affiliations:** Institute of Philosophy, Freie Universität Berlin, Berlin, Germany

**Keywords:** Affect, Affective stasis, Affective plasticity, Standstill, Upheaval, Covid-19 pandemic

## Abstract

The Covid-19 pandemic put forth a new kind of affective exhaustion. Being forced to stay at home, diminish social interactions and reduce the scale of their everyday mobility, many people experienced boredom, sluggishness, and existential immobility. While state-imposed pandemic policies changed rapidly, everyday life remained strangely unmoving. A sense of being stuck unfurled―as if not only social life, but time itself had come to a halt. At the same time, there was a latent sense of tension and increased aggressiveness which became manifest not only in protests and riots, but also in the texture of everyday life. In this contribution, we argue that both of these states―the feeling of being stuck, and the feeling that this putative tranquility is nothing but the calm before a storm―can be conceptualized as affective stasis. Through a rearticulation of the ancient concept of stasis, we show that these two at first glance incongruous affective conditions are intricately entangled. In Ancient Greek, the term stasis meant “stand, standing, stance”. Being used in a wide variety of contexts―politics, navigation, sports, rhetoric, medicine, and others―stasis took on different meanings which can be semantically organized around two opposite poles: one is the total absence of motion, and the other is an event of radical and often violent social and political change. Drawing on affect theory, phenomenology, and ancient Greek semantics, we propose affective stasis as a novel conceptual framework for political phenomenology.

## Introduction: on being stuck

The Covid-19 pandemic put forth a strange new kind of affective exhaustion. Lockdowns and strict contact restrictions were something most citizens in Central and Western Europe had never experienced before. Not surprisingly, the restrictions affected social classes in unequal ways: Whereas most blue-collar workers had no choice but to leave home if they did not want to lose their jobs, white collar work was largely relocated to the domestic sphere. Being forced to stay inside, diminish social interactions and drastically reduce the scope of their everyday mobility, those who worked from home tended to experience a blurry disarray of boredom, sluggishness and existential strain. While state-imposed pandemic policies changed frequently, everyday life remained strangely unmoving. A sense of being stuck unfurled―as if not only social life, but time itself had come to a halt. At the same time, however, there was also a latent sense of tension and growing tetchiness that seemed to creep through the social fabric as a whole. Domestic violence rose to a new level (see Anderberg et al., 2021). And the streets were regularly occupied by unsettling scenes of protest. The longer the pandemic crisis lasted, the more one could sense an atmosphere of irritability and aggressiveness―on social media, in politics, in everyday micro-encounters. Underneath the imposed calm, a mixture of more uncanny affects seemed to be accumulating.

In this article, we will argue that both these states―the feeling of being stuck on the one hand, and the feeling that this tranquility might be nothing but the deceptive calm before a storm on the other hand―can be conceptualized as *affective stasis*. Through a rearticulation of the ancient notion of stasis, we will demonstrate that these two at first glance incongruous affective conditions are, in fact, intricately related to one another. In Ancient Greek, the term stasis basically meant “stand, standing, stance” (Kennedy, 2011, 98). Being used in a wide variety of contexts―politics in the first place, but also sports, rhetoric, and medicine―stasis took on different meanings which can be semantically organized around two opposite poles: the first one being characterized through the total absence of motion, and the second one being characterized through an immediate event of radical―and often violent―political change (Bernhardt, 2021a, 13–15). Drawing on social phenomenology, cultural affect studies and ancient Greek semantics, our aim is to introduce affective stasis as a new conceptual framework for thinking through the affective conditions of social life under the specific circumstances of the Covid crisis. We do so, in the present paper, by showing that the concept of affective stasis helps us to elucidate the affective climate of the pandemic crisis in which stagnation and flux, inertia and change came to intertwine in peculiar ways. Over and above elucidating a dimension of the affective experience of pandemic life, we hope to contribute to a contemporary political phenomenology by suggesting “affective stasis” as an analytical lens for considering a range of affective conditions with considerable political significance (cf. Bernhardt, 2021a).

Most of this article was written in the second half of 2021, while the pandemic crisis was still ongoing, with ever new waves of infections spreading around the globe. Meanwhile, the situation has changed somewhat―at least in most European countries. There are signs indicating that the pandemic has passed its zenith. Even governments which ran rather restrictive Covid policies, for example in France and in Germany, are successively relaxing the measures which have curtailed social life for so long. While the immediate sense of emergency is fading and the contours of a post-pandemic landscape hesitantly emerge, the feeling of being stuck is giving way to other sentiments, moods and emotions.

We deem it important to stress that our account does not claim to capture *the* affective experience of the pandemic. In fact, we doubt that there is such an experience in the singular, even if one only considers one country, region or specific group. Depending on their positionality―which, among other things, involves race, class, gender, religion, political stance, occupational status and cultural situatedness―people experienced the Covid-19 pandemic in manifold ways. The hardships of the pandemic were distributed differentially and―as usually in times of crisis―largely to the detriment of those who were already in unprivileged positions before. It clearly made a difference whether one was working on the assembly line or at university, delivering food by bike or treating patients in a hospital, whether one had to care for children or not, facing financial troubles, living in a house with garden or in a small flat, and so on (cf. Protevi, 2022). All of this fed into the ways in which people felt during the pandemic.^1^ Obviously, this applies also to our account which is shaped by our own positionality and, thus, inevitably prone to middle-class bias. Nevertheless, we do believe that there are at least some affective experiences which a great number of people shared during the pandemic, particularly in regard to the unprecedented situation of lockdown. Not in spite of, but because it was written from a *situated* perspective,^2^ right in the middle of the pandemic, under the immediate impact of social confinement, our account might help in coming to terms with the pandemic crisis since it aims at capturing something that other approaches easily miss: the latent, subtle and elusive undercurrents of affective life that are hardly grasped within conceptual frameworks which focus solely on fully developed emotions, i.e. the level of affective experience which has already become nameable, distinct and unambiguous. This is why the notion of affect―especially as it figures in the theoretical tradition that links Spinoza via Deleuze with contemporary cultural theorists such as Brian Massumi, John Protevi and others―plays such an important role in our framework (see Gregg & Seigworth 2010; Massumi, 2002; Protevi, 2009; Slaby & von Scheve, 2019). With Lauren Berlant, we believe “that the present is perceived, first, *affectively* [emphasis added]: the present is what makes itself present to us before it becomes anything else, such as an orchestrated collective event or an epoch on which we can look back.” (Berlant, 2011, 3) Our attempt to think through the pandemic crisis from within this crisis thus involves a special attention for the lurking, tacit and covert dimensions of the affective that cannot be readily grasped in terms of fully shaped out emotions or distinct mental states. By proposing affective stasis as a concept, we seek to expand the conceptual repertoire and analytical toolbox of phenomenological and scientific emotion research in a direction that might help us get to the messy bottom of the pandemic crisis―which is still far from being an epoch on which we can indifferently look back. Over and above our focus on the pandemic, we also want to propose affective stasis as a conceptual perspective to orient work in phenomenology and affect theory towards a so far under-appreciated area of affective conditions. These are modes of stunted vitality that widely ramify through a person’s practical perspective, especially with regard to affective and practical capacities for social connection and relatedness.

## In search of new words: languishing

In the very beginning of the pandemic, when people where still struggling to apprehend what was going on, the feelings it evoked nevertheless seemed relatively tangible. There was the initial shock, followed by a widely shared sense of disbelief, there was fear, there was anxiety. Then, at least for many, there was grief. Being able to name one’s feelings―fear, anxiety, grief―tends to make it easier to handle them. After that very first weeks and months, however, it got much more difficult to find such names. The more the pandemic crisis came to establish itself as the “new normal”, the blurrier got its affective contours. The question “How are you?” for many was not easy to answer. The available emotional vocabulary seemed insufficient to describe this mushy state. The same applied to the psychological thesaurus: One was drooping, but it was not enough to call it a depression. One was weary, but not exhausted enough to let this weariness pass as chronic fatigue. No single term seemed apt to capture the core of this affective experience whose main characteristic seemed to be precisely that it felt like no experience at all. It wasn’t fear, grief, or any distinct emotion, but rather something like a general *affective climate*, a latent atmosphere, which only partially became manifest in clearly identifiable ways.

Not surprisingly, this situation incited a search for new words that would allow to pin down this blurred experience. One linguistic label was proposed by organizational psychologist Adam Grant. In the spring of 2021, right in the middle of the “third wave” of the pandemic, he published an article in the *New York Times* under the headline “There’s a Name for the Blah You’re Feeling: It’s Called Languishing” (Grant, 2021, April 19). Already from the first lines, it becomes clear that the “blah” Grant refers to is not an individual or even idiosyncratic experience, but rather an affective condition widely shared:

At first, I didn’t recognize the symptoms that we all had in common. Friends mentioned that they were having trouble concentrating. Colleagues reported that even with vaccines on the horizon, they weren’t excited about 2021. A family member was staying up late to watch “National Treasure” again even though she knows the movie by heart. And instead of bouncing out of bed at 6 a.m., I was lying there until 7, playing Words with Friends.

It wasn’t burnout — we still had energy. It wasn’t depression — we didn’t feel hopeless. We just felt somewhat joyless and aimless. It turns out there’s a name for that: languishing. (2021, April 19)

“Languishing” was not created during the pandemic, although its excellent fit could let one presume as much. It is an old word whose use in everyday language had of late gone out of fashion. According to the Oxford English Dictionary, languishing means

to weaken, wither, or become faint; […] [t]o live in an oppressive or dispiriting place, situation, or condition. […] To fail to make progress; to be unsuccessful. […] To droop in spirits; to pine or brood […]. […] To waste away with longing for; to yearn (to do something). […] Of an activity or emotion: to grow slack, lose vigour or intensity. (Oxford University Press, n.d.)

These descriptions seem apt to capture the general affective make-up during lockdown. In his *New York Times* article, Grant himself describes languishing as “a sense of stagnation and emptiness. It feels as if you’re muddling through your days, looking at your life through a foggy windshield.” According to Grant, this “dwindling of drive” often comes unnoticed; it is not a condition that suddenly befalls you, but one you slowly slide into. If grief was the dominant emotion of 2020, then languishing, Grant concludes, “might be the dominant emotion of 2021” (2021, April 19).

The term languishing captures important features of what it felt like to live under the conditions of a constantly elongating state of emergency. It accounts for the inertia and existential anti-flux. At the same time, however, it misses an important point: People were not only more tired, but also more irritable than usual. Inside homes, there was not only more drooping and brooding, but also more shouting and nagging. And in the few public spaces which remained accessible during lockdown―supermarkets, public transport, the queue in front of the bakery―more and more often one could see people fighting and clashing with each other. Languishing, thus, only captures one side of the picture. It accounts for the *low-intensity* affects, the feelings of being stuck, but it fails to apprehend its *high-intensity* flipside. Of course, there was exhaustion, but, at the same time and in its shadow, there was also the looming inkling that at any moment things (including oneself) might explode. Sometimes, it felt as if there was nothing but a thin membrane between exhaustion and explosion, total ossification and incalculable upheaval. Grandmaster Flash’s 1982 message―“Don’t push me / Coz I’m close to the edge / I’m trying not to lose my head” (Fletcher et al., 1982)―can be conceived of as 2021’s hidden refrain. Enduring under pandemic conditions involved living permanently in the vicinity of this edge.

In fact, this might be one hidden reason why people became so exhausted during the pandemic: It’s energy-sapping to be constantly balancing on the edge of a cliff. The attempt not to lose one’s head, nerves or temper afforded a permanent form of affective labor, which for the most part went unnoticed (not only by the social environment, but also by those who performed it). The notion of languishing captures the feeling of being stuck, but it misses its tetchy flipside. It neither explains the emotional edginess nor the latent affects that unaware labor was supposed to deflect. Another concept is needed which provides a more comprehensive understanding of the affective climate during the pandemic and how its two sides relate to one another. We believe that affective stasis is more apt to meet this demand.

## Revisiting ancient semantics: Stasis

Unlike other ancient Greek words (for example, gymnastics, politics, or architecture) the term stasis never really made it into the everyday lexicon of modern-day English. You will hardly hear it in ordinary conversations or read it in the newspapers. The only domain in which the word has been able to gain a firm foothold in contemporary English is the one of medicine. In medical terminology, stasis basically refers to a “stagnation or stoppage of the circulation of any of the fluids of the body” (Oxford University Press, n.d.) which normally (that is, when the vital functions are running smoothly) are in motion. Not every form of stagnation or immobility, thus, is to be understood as stasis, but only the immobility of something that is *supposed* to be constantly moving. Depending on the type of bodily fluid, medicine distinguishes between different forms of stasis, such as haemostasis, or lymphatic stasis. Such congestions can be dangerous and pathogenic. Prolonged constipation, for example, can result in a gradual self-poisoning of the body. Outside the relatively well-delineated domain of medicine, however, the word stasis is rarely used in our days.

In ancient Greece, the situation was different. Depending on the context, stasis (στάσις)―the plural being staseis (στάσεις)―could take on a wide array of meanings, which, again depending on the context, branched out into an increasingly finely differentiated semantic field. To get a first impression of this field, let us have a look at one of the standard school lexicons of ancient Greek, the so-called “Little Liddell” (Liddell & Scott, 1906):

**στάσις** […] *a standing, the posture of standing*. 2. *a position, post, station: a point of the compass* […] 3. *the state* or *condition in which a person is*, Lat. *status*. II. a *party, company*: esp. *a party formed for political purposes*, *a faction, party*. 2. *sedition, faction.*

The most decisive lexicon of ancient Greek, the so-called Liddell-Scott-Jones―sometimes also referred to as “Great Scott” or simply LSJ―adds more details to the picture, yet also makes it more difficult to overlook (cf. Liddell et al., 1940).^3^ Under the basic meaning “*placing, setting*”, reference is made to the act of erecting a statue, “*standing stone, pillar*”, and “*erection, building*” in general. As sub-items of the meaning “*standing still, stationariness*” the LSJ lists “*standing, stature*”―the opposites being φορά and κίνησις both of which can be translated with “movement” ―, “*rest*”, “*constipation*”, and “*sluggishness*”. The latter term already indicates that stasis was not only used for the immobility of physical bodies (like standing stones or pillars), but also for a form of immobility which is genuinely affective. Particularly wide-ranging are the contexts in which the word was used to denote “*the place in which one stands or should stand, position, posture, station*”: In navigation stasis could mean the “*position in relation to the compass*”, in rhetoric the “*position, opinion* of a philosopher”, in ancient sports it could refer to “a boxer’s *position*”, and in the realm of law to the “*position* taken up by a litigant”. Outside these specific contexts, stasis could also generally mean the “*state of affairs*” or the “*position, state, condition* of a person […]; esp. of moral, social, political *position*”. Furthermore, stasis could also mean “*party, company, band*”, especially a “*party formed for seditious purposes*”, “*faction, sedition, discord*”, and “*division, dissent*” (Liddell et al., 1940).

Given how broad this semantic field is, it is difficult to make sense of it at first. What holds all these different meanings of stasis together? Is there something like a common denominator? The direction of an answer is pointed out by the consideration that stasis at the same time is both a *positional term* and a *postural term*. It does not only answer to the question *where* a particular body stands, but also to the question *how* this body is standing. All usages in one sense or another refer to the position of bodies in a specific space or environment―no matter whether this is a courtroom, a boxing ring, a political assembly or the social fabric as whole (stasis in the sense of “status”). Stasis, thus, is always about *situatedness* in a comprehensive sense, not only understood as a spatial position, but also as social, political, moral, and affective one. Conceptualizing stasis as situatedness involves thinking through the ways in which position and posture, standing, status and stance, are interdependent. It involves an “ecological” understanding of the relation between bodies and spaces. The ancient notion of stasis seems to indicate that the potentialities of a body―its agency and possibilities of being―are pre-structured by the position it occupies in a specific environment and its relation to other bodies. This implies de-potentiating the modern notion of agency as a capacity that all people can freely make use of in equal ways. The ancient understanding of stasis rather suggests that your agential and existential possibilities―what you can do and be―strongly depends on where and how you are standing. Your stasis suggests certain (re)actions and precludes certain others.

To give some examples: In boxing, a southpaw is more likely to attack with her right arm; that means that in her basic stance or fighting position certain moves are more preordained than others. The same holds true for a social position, a philosophical opinion, or the position taken up by a litigant in a legal dispute: In certain spaces, you can often already tell from the bodily posture of a person if she is rich or poor. A Platonist philosopher will hardly produce materialistic arguments. And a defense lawyer who represents the standpoint that her client is innocent, will unlikely pursue the strategy of claiming diminished culpability. Where you stand and how you are standing influences which moves you will make and which not. This comprehensive idea of situatedness is well-captured in the English translation of stasis as “stand, standing, stance” (Kennedy, 2011, 98). In fact, the ancient Greek word “stasis” and the English words “standing” and “stance” etymologically share the same root (see Frisk 1960a, 1960b).

Stasis, however, is not only a positional and postural term, but also, and even in the first place a *political* one. The political sphere―that is, the material and imaginary space of the polis―was the realm in which the notion of stasis became particularly relevant (see Agamben 2015; Börm, 2019; Gehrke, 1985; Hansen 2004; Hansen, 2006; Loraux, 1997; Ober, 2003). This comes into view when focusing on its meaning as faction, sedition, discord, and dissent. For, contrary to their idealized image, the Greek poleis were by no means havens of harmony. On the contrary, there were frequent internal conflicts and fissions that threatened to destroy the unity of the polis from within. It was characteristic of these conflicts that two hostile groups were facing off, competing for political power (Börm, 2019, 13). In the ancient political vocabulary, stasis referred not only to these factions, but also to the disintegration of the polis itself, its polarization and falling apart into two factions, as well as to the conflicts that arose from this division. Often, these conflicts grew extremely violent. Stasis became a word for uprising, revolution and civil war. In the ancient world, such upheavals were widespread. According to Josiah Ober, “stasis was a terror that perpetually stalked Greek political landscape” (2003, 251). In the same vein, ancient historians Mogens Herman Hansen and Hans-Joachim Gehrke refer to stasis as an “everyday phenomenon” (Hansen, 2004, 126) and “essential phenomenon of Greek history” (Gehrke, 1985, 266). The nature of these conflicts and competing factions Hansen explains as follows:

To judge from our sources, most *poleis* were split into two rival *poleis*, one of the rich, who supported oligarchy, and one of the poor, who preferred democracy. The rival parties could also be different ethnic groups living side by side in the same *polis* […]. Or the community could be polarised around two rival groups of rich contending for power. In all those cases the purpose of both groups was to control and, if necessary, reform the institutions of the city. The result was almost constant political tension, which often led to civil war, in which every group was ready to work hand in hand with a like-minded group in a neighbouring city or in one of the powerful cities that led the shifting alliances of *poleis*. The members of both groups were therefore prepared to sacrifice the independence and autonomy of their city if, in return, they could keep or win power in the *polis*. […] *Stasis* always means a group that wants to preserve or obtain power by deceit or violence, i.e., a revolutionary group, never a political group operating within the constitutional framework of the city-state, i.e., what we call a political party. (Hansen, 2006, 125)

The willingness to sacrifice the autonomy of the polis and to submit to a foreign power rather than surrender to the internal opponent indicates the relentlessness with which the struggle for supremacy in the polis was waged.

Taking the polis as central reference, the various ways in which the word stasis was used in ancient Greek can be arranged into the following sequence:

The word *stasis* actually means ‘stance’; but it underwent shifts of meaning as follows: (1) stance, (2) standpoint, (3) group of people with the same standpoint, (4) in the plural: two or more groups with opposing standpoints, (5) the split between groups, and (6) civil war. (Hansen, 2006, 125)

The political meaning of stasis is not independent from its usage as positional and postural term: The unwillingness to give up one’s standpoint and take a different stance―in other words, the foreclosure of *movement*―was one of the reasons why conflicts within the polis frequently grew into civil wars. Construed in such way, the sequence almost conveys the impression of a certain historical consequence―as if the polis was inevitably condemned, as it were, to go through this circle again and again. One should be careful though not to jump to conclusions. The fact that the ancient poleis were *frequently* torn by internal discord provides no sufficient reason to assume that they were *necessarily* doomed to sink into chaos. The above sequence thus by no means reflects a teleological necessity. Instead of presuming some general law of decay in the vein of Spengler (1998), the ancient political phenomenon of stasis rather has to be analyzed in its case-specific contexts which as such remain changeable and contingent. History does not follow pre-scripted patterns―neither those of progress nor those of decay.

We now have got an idea of what stasis meant in the ancient Greek world. But what exactly does *affective* stasis mean when we transfer this notion to the historical present? The ancient semantics alone do not amount to a concept apt for present purposes. Accordingly, in the following section we will sketch out how one might forge the semantic substance of the ancient term into a conceptual framework that helps us to better understand the pandemic crisis.

## Towards a conceptual framework: affective stasis

As indicated, affective stasis does not denote a single emotion, but a broader affective condition. In this and the next section, we delineate the contours of affective stasis as a conceptual perspective for a contemporary political phenomenology. As a working definition, we propose to understand this condition as characterized by a general *lack of affective plasticity*. Affective plasticity refers to the ability to affect and be affected in heterogeneous and ever new ways.^4^ It encompasses various dimensions of affectability, among them the capacity to become affectively attuned to other people and to resonate affectively with their expressed affectivity, bodily comportment and demeanor. On a more basic level, affective plasticity refers to the capacity to become affectively engaged at all: a vital responsiveness to one’s surroundings. It is clear that deficiencies in this capacity have significant negative ramifications for a person’s being-in-the-world in general.

Although such a marked lack of affective plasticity can be described in psychological terms, it is important to pay tribute to the semantic traces which draw a close line between the notion of stasis and the political. Affective stasis does not fall from the sky―and it is not a mere physical affliction that might come and go according to organic causes. Rather, it has to be understood as resulting from particular social and political conditions. There are governmental technologies and institutional arrangements which, wittingly or unwittingly, produce affective stasis. Think, for example, of prisons, refugee camps or detention centers.^5^ The political production of affective stasis, however, can also come along in much more subtle forms which work without any visible form of coercion (cf. for examples Bernhardt 2021a, 18–31). In this regard, we think that some of the public measures imposed to contain the Covid-19-pandemic had a similar effect: in reducing the variety and intensity of social life and by confining individuals spatially, measures such as social distancing, lockdowns, public access restrictions and related procedures served as fertile ground for the emergence and instantiation of affective stasis.

Conceptually, affective stasis is clamped between two opposite poles: *immobility* on one side and *disruption* on the other. During the pandemic the societal spheres of medicine and politics became particularly important. Indeed, exactly these two domains are also particularly helpful when it comes to illuminating this polarity. They mark the opposite directions that define the scope of what stasis can be: Congestion or stagnation of fluids within a single body (medicine), taking a stand, hardening of the fronts, revolt and civil strife within a community composed of many individual bodies (politics). The movement from one pole to the other does not take place smoothly though, in a gentle glide, but discontinuously with an abrupt jolt. Between immobility and disruption, standstill and upheaval, there is a tipping point, a critical threshold. It marks the point at which things can no longer just go on as they are and the “state of affairs” (which is also one ancient meaning of stasis) abruptly changes. Part of the fascination of the concept of affective stasis is due to the question of this threshold: At which point, prompted by which factors, does an immobilizing, sluggish condition tip over into its intensive, eruptive opposite? Before we attempt to answer this question, it is important that we get clearer on the two contrasting dimensions of affective stasis.

Both poles come along with specific affective qualities. The general “feel” of the immobility-pole can be described in terms of deprivation: as loss of intensity, vigor or emotional fluency. The immobility-pole is not only connected to feelings of boredom and stagnation but can also take the form of idle complacency or blatant frustration. When tending to this pole, affective stasis acts passivating and stiffening. In this regard, affective stasis resembles the condition conceptualized by Frantz Fanon as “affective ankylosis” (1967, 122). Like stasis, ankylosis is originally an ancient Greek term which today is primarily used in medicine. Within this sphere, it refers to the stiffening of a joint or the coalescence of two bones (Oxford University Press, n.d.). Both notions thus refer to a condition in which something that is supposed to be moving―lymph, blood, the hinge of an elbow, a finger joint―loses its mobility. Fanon transposes the term from the organic to the psychological, in order to indicate an affliction that parallels the stiffening of joints in the affective and cognitive realms: a rigidification of psychological dispositions that give rise to mechanical routines spanning perceptual, emotive and judgmental capacities. Affective plasticity refers to the fact that a person’s emotional repertoire is usually fluid, variable and capable of expanding to accommodate new experiences, but in a state of ankylosis it becomes fixated upon a small range of schematic responses, mechanically triggered by typical prompts, impervious to variation and blind to contrary evidence. As Alia Al-Saji explains Fanon’s use of the term, affective ankylosis refers especially to the immobility of racializing affect which “is not only frozen in its response but repetitive in its form” (2014, 141). Again, it is important to emphasize that this “stuck affectivity” (Blickstein, 2019, 158) does not simply arrive out of the blue. Affects of racialization are constituted by long-term sociogenetic processes whose emergence is concomitant with the formation of colonial modernity itself.

The affective qualities connected to the other side, the pole of disruption, are more difficult to grasp since the feelings connected to it for the most part remain latent and vague. Initially, much like an approaching storm, the coming disruption can only be presumed to occur, based on indirect hints such as subtle changes in the affective atmosphere. One feels that something is “in the air” without being able to precisely pin it down. It is not the disruptive event itself, but rather that which comes right before it: a sudden tension, a nervous twitch in the eye, an electric crackle, a fist closing. Even if this affective state is experienced as intensity-increasing, it still has something to do with a lack of affective plasticity: The polarisation which precedes the disruptive event―no matter whether this refers to civil wars in ancient poleis or to violent clashes between anti-vaxxers and police forces nowadays―results from an antagonistic social constellation or political impasse in which neither side is willing to move. The obduracy characteristic of enmity and hatred also points to a lack of affective plasticity. Although immobility and disruption feel differently, they are equally induced by a lack of affective plasticity. Phenomenologically, this state can be described in terms of a deadlocked tension, a charged condition perpetually at the verge of an outburst, but usually stopping just short of an actual eruption (of vigorously expressed anger or even manifest violence). The difference between the two poles can also be articulated in terms of temporality. Whereas the immobility-pole is related to slowdown and stagnation, the disruption-pole is related to a sense of precipitous (and often catastrophic) acceleration. Affective stasis, thus, encompasses both: the dull routine in which it feels like nothing is changing at all, and a tensed anticipation of a sudden disruption in which all things seem about to be overthrown.^6^

It is precisely this polarity which makes the concept well-suited for capturing the general affective climate during the pandemic. While it is true that the virus compelled many to unlearn old habits, it is also true that the new routines and behaviors we developed instead very soon became stifling and sour. Even for the more privileged, domestic space under lockdown turned into a space of confinement. Being confined to the same rooms with the same individuals, following the same routines day after day, bereft of new experiences and contingent social encounters (see Alloa 2020), amounted to the feeling of being stuck both in space and time. With the repeated imposition of similar restrictions, there came not only feelings of exhaustion and powerlessness, but also latent aggressiveness and frustration. On the political and social level, the virus thereby brought forth new fissions in society and, at the same time, intensified those that were already present. Increasingly, news images of overcrowded intensive care units were joined by images of collective protests and violent riots in the streets. While social life slowed down, political disintegration accelerated. Conceiving of the pandemic crisis as affective stasis involves conceiving of immobility and disruption as being two sides of the same coin.

## Critical thresholds: between standstill and upheaval

How exactly do these two sides relate to each other? According to which inner logic does a state of immobility turn into a disruptive event? What we have developed so far suggests that affective stasis is organized around an elusive tipping point at which the stagnating sloth of its immobility-pole gives way to a tension-riddled state of high intensity that lets those in its thrall hover on the verge of an aggressive outburst. Congestion, stagnation, standstill eventually gives rise―without this event having any compelling necessity―to an abrupt rupture that violently shakes the individual or social body from within and throws it into turmoil. When exactly the critical threshold is reached is difficult to foresee. For instance, the revolutionary change climate activists and other civil groups started to hope for at the beginning of the pandemic did not occur (see Schütze et al., 2022). Due to this logic―which makes it delicate to predict the tipping point―affective stasis is accompanied by an irreducible ambiguousness.

A phenomenological consideration may help us to venture a step further. Affective stasis encompasses a felt dimension. What is it about the felt quality of affective stasis that precipitates its transformation into a phase of aggressive tension? A simple consideration that also fits the pandemic situation goes as follows: As opposed to an instance of “regular” sluggishness―for example, as resulting from physical or mental exhaustion―affective stasis does not simply result from a lack of vital energy. Rather, it ensues from a forced immobilization, externally imposed―a blockage and foreclosure of affective flux―so that some element of affective energy is retained, but for a certain span of time so rigorously obstructed that it can no longer manifest as a feature of the overall felt condition. A peculiar mixture results: a condition of surface immobility and languidness that nevertheless retains an underside of suspended vitality. If this consideration is on the right track, one could say that there is something in the very quality of such imposed affective stagnation that lets us approach, stepwise and without much active noticing, a transition into a state of heightened tension and intensity. Such a phase-transition is particularly likely to unfold in case the conditions of an imposed restriction of vitality continue to obtain over longer periods. Yet, at which point this transition will actually be felt, at which point this subcutaneous tension surfaces so that it begins to figure in experience, if only marginally, is hard to say, and will likely vary from case to case. To be clear: affective stasis, as we envision the notion, is not solely and not predominantly a concept meant to elucidate an *experiential* condition, let alone an instance of mere subjective feeling. Rather, it refers to a broader condition of socio-historical situatedness which, at least in some of its instances, gives rise to, or goes along with, consciously felt manifestations. In this regard, one might consider it an instance of what Heidegger has described as *Befindlichkeit* (“findingness” or disposedness; see Slaby 2017). A good way to concretize this ontological notion is by way of the concept of atmosphere. Affective stasis can manifest atmospherically, for example as a tangible tension hanging in the air, a peculiar charge besetting domestic as well as public spaces (supermarkets, commuter trains, bus stations), creating a sense of imminent threat or panic among passers-by.^7^

Concerning the pandemic, the to and fro between tension and sloth was additionally fostered by the features of the SARS-CoV-2-virus itself. In the first weeks and months of the pandemic, we knew that the virus was able to kill people, but on the other hand we also heard of cases in which people got infected and hardly suffered from any symptoms at all. Having to endure social restrictions on the one hand definitely was aggravating and had its price, but on the other hand these restrictions did not deem totally unbearable. A lot of things did change since the virus began to dominate our daily lives, but on the other hand there were days on which it appeared like nothing had changed at all. A revolutionary change did not occur. From a certain point on, all most people hoped for was the return of the status quo ante. If SARS-CoV-2 was as deadly as say Ebola, one can assume that it would have prompted a less ambiguous response in terms of existential urgency. In this regard, the Covid-19 pandemic is different from other existential threats: in case of a Tsunami or a terror attack people switch into survival mode and run. For the most part, the pandemic did not have this drastic effect. On the contrary, either in the news or in one’s direct social environment, one could witness an opposite effect: As long as the virus did not severely affect oneself, a loved one or a person in one’s direct proximity, there remained the astonishing freedom to downplay its dangerousness or even deny its existence. But even for those who did not succumb to conspiracy theories or other forms of distortion and denial, the actual threat often appeared abstract and distant. Reasonable measures such as social distancing and contact restrictions in their daily enactment therefore often retained an air of arbitrariness. Despite better knowledge, very little in our day-to-day routines conveyed to us the actual presence of danger. For many people the situation thus tended to assume a sense of estrangement and even unreality. This sense of unreality, however, did not prevent the feeling of being stuck from taking hold more and more. We think that this lack of urgency has played a role in the overall condition of affective stasis under Covid-19, among other factors which have jointly accumulated to the impression of sluggishness, boredom, lack of vigor and vital force. One other such factor is worth discussing, as we deem it particularly central.

For a majority of people in Central and Western Europe, the pandemic has markedly lowered the temperature of social life for quite some time. The various restrictions and distancing measures diminished the frequency and intensity of social interaction in general, and in particular inhibited many of those interactions that are of a festive, convivial, non-mandatory kind.^8^ It is exactly those social occasions that play a strong role in bestowing meaning and excitement to everyday existence. They make up a tangible “social substance”, an emotional energy that is crucial to the flourishing of individuals and collectives. In light of this, it is not far-fetched to assume that the quality of interpersonal relatedness was considerably impoverished during the pandemic. The sustained lack of festive togetherness contributed to the imposed inertia, and thus played a central role in the generation and maintenance of affective stasis. Lacking opportunities to connect affectively led to a suspension or even unlearning of social habits and behaviors related to conviviality and affectionate being-together. As with most phenomena in this pandemic, this was a slow, partial, piece-by-piece development, sometimes hardly noticeable and inconspicuous: instead of a radical rupture there was a gradual waning of substantive sociality and vitality from our lives.

However, concerning this point it might be the case that the pandemic crisis only brought to light a more general tendency: Even before the pandemic struck in early 2020, the intensity and range of convivial, non-utilitarian sociality in late-capitalist societies had already been in decline. Accordingly, it could well be that the Covid crisis only focused and amplified a sentiment that had been much longer in the making: a broader sense of stagnation, of impasse, of cultural and spiritual exhaustion, accompanied by a hardening of social relations (cf. for the situation in the United States Putnam 2001). We think that the concept of affective stasis could further the development of an analytic of such conditions, aligning phenomenological considerations of the affective dimension of substantive sociality and its deficient modes with work in cultural theory on the affective landscapes of neoliberal late modernity. In this regard, the political phenomenology of affective stasis that we envision meshes well with, for instance, Lauren Berlant’s (2011) work on “cruel optimism”, Mark Fisher’s (2009) outlook on “capitalist realism”, Ann Cvetkovitch’s (2012) observations of depression as a “public feeling”, or Sianne Ngai’s (2005) chartings of “ugly feelings” that pervade the everyday. A central task for such an analytic will be the assessment of the extent to which these prevailing affective conditions either preclude or still leave room for the possibility of political agency capable of addressing the polymorphous global crises of today in meaningful ways.

## Conclusion and outlook

Based on and inspired by Ancient Greek semantics, we have proposed the notion of affective stasis as conceptual framework for analyzing some dimensions of affective life during the pandemic crisis, in particular those that might easily be overlooked from perspectives focusing on fully shaped out, nameable emotions and feelings. By dwelling on the elusive undercurrents of affective experience, the notion of affective stasis can complement the conceptual repertoire of emotion research in ways that contribute to a better understanding of the altered affective conditions the pandemic has instigated. In this conclusion, we highlight three aspects of our account we deem particularly promising for further development of our conceptual proposal and for bringing out its potential for a broader application over and above the situation of the Covid-19-pandemic.

(1) The concept of affective stasis helps us to carve out the link between the feeling of being stuck and its tetchy affective backside. At the same time, it directs analytical attention towards the tipping point at which an enduring state of affective immobility resulting from an externally imposed impasse turns into an event of disruptive, tension-riddled change. While it is true that lockdowns and other state-imposed restrictions aimed at controlling social movement and interaction, it is also true that this form of control was rather means than purpose. In other words, political authorities hazarded the consequences that gave rise to affective stasis, but it would be misguided to assume that the restrictive measures were intentionally designed to produce an affective climate of sloth and inertia. But there are other instances where the production of affective stasis is a crucial part of governmental technology rather than an unwanted side effect. Studies on migrant affects and institutions like refugee camps, prisons and detention centers, for example, could benefit from integrating the concept of affective stasis into their analytical toolbox. In fact, we do believe that emotion research in general deals with a range of topics whose affective dynamics evolve around a polarity comparable to the one we described as characteristic for affective stasis.

(2) Central to affective stasis is a lack of affective plasticity. During the pandemic crisis the dwindling of affective plasticity was remarkably doubled by a parallel dwindling of social vitality, convivial encounters and festive togetherness. The latter can be conceived of as rich modes of social relationality which belong to the same sphere as the practices of reciprocal exchange Marcel Mauss described in his famous essay *The Gift* (1966). We deem it promising to follow this line further, not just for bringing out the peculiar affective texture of life during the pandemic, but also for enriching the overall understanding of the social dimension of affectivity. Theories of gift exchange highlight forms of “enhanced” sociality where social relatedness expands well beyond the experiential orientation of interacting agents to include a rich repertoire of gestures, bodily styles and orientations, greeting practices and certainly also material exchange in various registers. The social distancing measures implemented during the pandemic have considerably curbed much of this richer sociality; we think that the resulting condition of severely stunted affective vitality is indicative of the significance of this oft-neglected dimension.

(3) Our proposal emphasizes the importance of relating the affective to the political. We have sketched the contours of a framework that circles in on affective conditions that supervene not only on features internal to agents or to interacting individuals, but encompass parts of the wider socio-political context in which affective experiences are situated. Affective stasis is more than a modality of felt experience, and, as the ancient Greek semantics of the term reminds us, it is connected to aspects of political constellations: for instance, to a situation of standoff between different factions in society whose respective standpoints have suddenly or gradually hardened into an antagonistic rift. Affective stasis in the political realm concerns situations in which a dangerous tension builds up between competing camps to a point where an open conflict seems ready to erupt at any moment. Therefore, we propose affective stasis as a diagnostic angle geared to political constellations and to the mindsets and affective orientations of those individuals that find themselves positioned in these constellations. This means that deploying this concept can only be productive if it is combined with a detailed analysis of the particular socio-political situation at hand; it requires a lot of detail on the nature of a given conflict, on the contesting factions and the history of the particular community and its various conflicts, alliances and dynamics of social change over time. The combination of a theoretical angle with such a broader diagnostic perspective makes this framework appealing outside the specialist realm of the philosophy of mind and emotion. Accordingly, we consider our proposal to be a contribution to a contemporary political phenomenology.

With the preceding analysis, we hope to have kindled broader interest in the notion of affective stasis and to have motivated efforts to further elucidate the notion and apply the associated conceptual perspective to other historical and contemporary constellations. As global crises proliferate, it is important to expand the conceptual repertoire of philosophy towards notions that originate from the human experience of political impasse. Complex affective conditions have to be front and center in such an endeavor.

## Footnotes

1 These distinctions were embedded into larger geographical contexts due to national differences in terms of government aid structures and social policies in general. For instance, in the United States people who became unemployed had to negotiate notoriously complex unemployment systems. In other countries, France and Germany for example, national governments provided financial aid to companies who retained employees even if salaries were reduced.

2 This “situated” understanding of scholarly practice can be well aligned with the phenomenological tradition following from Heidegger, and notably so where it is engaged with the affective texture of historical epochs and sedimentations which continue to inform the present (cf. Slaby, 2017 & 2020). Equally productively, it can draw on a recent strand of work in cultural affect studies that critically engages with the affective and atmospheric dimensions of late capitalist life worlds (see Berlant 2011, Cvetkovich, 2012, Ngai, 2005).

3 Cf. for a comprehensive picture the entire entry here (only the source references are omitted): ** στάσις** [ᾰ], εως, ἡ, (ἵστηµι) *placing, setting* […]; *erection* of a statue […]. **2**. *standing stone, pillar* […]. ** 3**. *erection, building* […]. **II.** (ἵστηµι A. IV) *weighing* […]. ** B.** (ἵσταµαι) *standing, stature* […]; *standing still, stationariness* […]; *rest*, as a category of the intelligible […]; *constipation* […]; [τοῦ αἵµατος] *sluggishness* […]. ** 2. ***the place in which one stands or should stand, position, posture, station* […]. ** b.*** position in relation to the compass* […]; *setting* of a wind from a quarter […]. ** c.** of planetary connexion […]. **d.** metaph., from a boxer’s *position* […]: hence, *position* taken up by a litigant […]; *issue* […]. ** e. ***position, opinion* of a philosopher […]. **3.*** position, state, condition* of a person […]; esp. of moral, social, political *position* […]; *state of affairs* […]. ** 4.** στάσις µελῶν […]. ** II. ***party, company, band* […]. ** III.** esp. *party formed for seditious purposes, faction* […]. ** 2. ***faction, sedition, discord* […]. **3.*** division, dissent* […]. ** 4.** metaph., τὰν ἀνέµων […]. **IV.** στάσεις, = τὰ πεφυκότα σπέρµατα […]. ** V. ***statute, decree* […]. (Liddell et al., 1940)

4 Our understanding of affective stasis as resulting from a lack of affective plasticity is partly influenced by Catherine Malabou’s notion of “destructive plasticity” (2012).

5 For an excellent account of life under the conditions of confinement from the perspective of critical phenomenology cf. Guenther, 2013.

6 The latter is also the reason why the condition of affective stasis ought not be confounded with an “ordinary” state of depression. While it is true that both affective stasis and depression come along with a felt immobility (what Viktor Emil von Gebsattel calls “vital inhibition” (von Gebsattel 1954, 138)), depression is not necessarily related to a sense of impending disruption.

7 The connections between the concepts affective stasis, *Befindlichkeit*, and atmosphere merits further elucidation. Concerning the notion of an atmosphere as an affect-theoretic concept, we have learned a lot from Friedlind Riedel’s succinct treatment (2019); an important phenomenological approach to emotions as atmospheric phenomena has been developed by Hermann Schmitz (see Schmitz et al., 2011).

8 This festive, convivial mode of sociality is intimately bound to the logics of reciprocal gift-exchange as it has been described by Marcel Mauss (1966) and Marcel Hénaff (2010). The logic of the gift does not only involve material goods, but also, and even more importantly, the exchange of social goods and formalized gestures such as invitations, greetings, compliments, courtesies or even something as fleeting as a mutual exchange of smiles among strangers on the street. The purpose of the gift is not to accumulate economic wealth, but to build up, maintain and strengthen social relationships. Following Mauss and Hénaff, one could say that precisely these kinds of interactions keep up the temperature of social life and prevent it from coming to a freezing halt. It would be worthwhile to explore a substantive concept of the social based on gift-exchange in more detail and in relation to phenomenological accounts of the interplay of affectivity and sociality (cf. Bernhardt, 2021b, 110–249).
